# SIMBA: a web tool for managing bacterial genome assembly generated by Ion PGM sequencing technology

**DOI:** 10.1186/s12859-016-1344-7

**Published:** 2016-12-15

**Authors:** Diego C. B. Mariano, Felipe L. Pereira, Edgar L. Aguiar, Letícia C. Oliveira, Leandro Benevides, Luís C. Guimarães, Edson L. Folador, Thiago J. Sousa, Preetam Ghosh, Debmalya Barh, Henrique C. P. Figueiredo, Artur Silva, Rommel T. J. Ramos, Vasco A. C. Azevedo

**Affiliations:** 1 Laboratory of Cellular and Molecular Genetics, Department of General Biology, Institute of Biological Sciences, Federal University of Minas Gerais, CEP 31270-901 Belo Horizonte, Minas Gerais Brazil; 2 National Reference Laboratory for Aquatic Animal Diseases of Ministry of Agriculture, Livestock and Food Supply, Federal University of Minas Gerais, CEP 31270-901 Belo Horizonte, Minas Gerais Brazil; 3 Centre for Genomics and Applied Gene Technology, Institute of Integrative Omics and Applied Biotechnology (IIOAB), Nonakuri, PurbaMedinipur WB-721172 India; 4 Department of Computer Science, Virginia Commonwealth University, Richmond, VA USA; 5 Institute of Biological Sciences, Federal University of Pará, Belém, Pará Brazil; 6 Federal University of Minas Gerais, Institute of Biological Sciences, Antônio Carlos 6627, Pampulha, 30161-970 Belo Horizonte, Minas Gerais Brazil

**Keywords:** Web tool, Genome assembly, Bacterial genome, Ion Torrent PGM, Genome finishing, Bioinformatics

## Abstract

**Background:**

The evolution of Next-Generation Sequencing (NGS) has considerably reduced the cost per sequenced-base, allowing a significant rise of sequencing projects, mainly in prokaryotes. However, the range of available NGS platforms requires different strategies and software to correctly assemble genomes. Different strategies are necessary to properly complete an assembly project, in addition to the installation or modification of various software. This requires users to have significant expertise in these software and command line scripting experience on Unix platforms, besides possessing the basic expertise on methodologies and techniques for genome assembly. These difficulties often delay the complete genome assembly projects.

**Results:**

In order to overcome this, we developed SIMBA (**SI**mple **M**anager for **B**acterial **A**ssemblies), a freely available web tool that integrates several component tools for assembling and finishing bacterial genomes. SIMBA provides a friendly and intuitive user interface so bioinformaticians, even with low computational expertise, can work under a centralized administrative control system of assemblies managed by the assembly center head. SIMBA guides the users to execute assembly process through simple and interactive pages. SIMBA workflow was divided in three modules: (i) projects: allows a general vision of genome sequencing projects, in addition to data quality analysis and data format conversions; (ii) assemblies: allows *de novo* assemblies with the software Mira, Minia, Newbler and SPAdes, also assembly quality validations using QUAST software; and (iii) curation: presents methods to finishing assemblies through tools for scaffolding contigs and close gaps. We also presented a case study that validated the efficacy of SIMBA to manage bacterial assemblies projects sequenced using Ion Torrent PGM.

**Conclusion:**

Besides to be a web tool for genome assembly, SIMBA is a complete genome assemblies project management system, which can be useful for managing of several projects in laboratories. SIMBA source code is available to download and install in local webservers at http://ufmg-simba.sourceforge.net.

## Background

Next-Generation Sequencing (NGS) has transformed the area of microbiology by enabling the sequencing of complete genomes of several prokaryote organisms [1]. This has led to the better understanding of epidemic outbreaks, improving diagnostic strategies, and the development of new drugs and vaccines. The high throughput data generated from NGS platforms are short reads, thus, the genome assembly is the process where short reads are then organized properly to represent the original genome [2].

The process of genome assembly can be split into three steps: (i) **data processing** - where the data quality is checked, handled and formatted to convert the data and prepare it for the next steps; (ii) **assembly** reads are overlapped (or de Bruijn graph structured) to build consensus sequences called contigs (*de novo* assembly); and (iii) **finishing**, which is the ordering of the contigs obtained in the previous step (scaffolding), and then determining the sequences that fit the gaps connecting one contig to the other (gap closing) [3, 4].

Scaffold and gaps closure steps can be performed *in silico* with help of a reference genome. There are several software to perform scaffolding, such as CONTIGuator [5], Mauve [6] or MapRepeat [7]. There are also strategies using mate-pair libraries, when the sequencing provides them. In addition, the approach of Whole-genome mapping (OpGen Inc, Gaithersburg, MD), or optical mapping, have been used to construct in vitro restriction maps that, when compared to *in silico* restriction maps of genome assemblies produced by the proprietary software MapSolver™ (OpGen Inc, Gaithersburg, MD), can scaffolding contigs. This technique has been considered able to produce assemblies with high accuracy, however depends of in vitro experiments and there are few free software to deal with these produced data [8].

In general, it is necessary to use many strategies, in addition to several tools, to finish assembly projects and obtain complete genome sequences [4]. This requires bioinformaticians to possess good knowledge in both software and hardware methodologies besides being trained on the techniques for genome assembly. Such trained users are hard to find and hence may delay the assembly projects besides increasing their costs. A remedy to these problems is the creation of user-friendly tools with good usability and minimum start-up time that can allow lay persons to complete the assembly projects.

In recent times, several software for genome assembly with good usability and simple installation processes are been proposed, such as the CLC Genomics Workbench (Qiagen, USA) and Lasergene Suite [9]. However, they are proprietary and only commercially available. Some works have shown the advantages of using free web tools in bioinformatics field [10, 11]. These web tools use different software that must be separately installed on a user’s machine to be functional. Often, the installation process and configuration of the different software modules in a server are complicated and require specific hardware requirements that may not be available to the user thereby making the whole process cumbersome. Though, after the installation common users can easily use the tool in a client browser in collaborative mode.

Hence, the use of user-friendly web tools, with easy installation and configuration, for assemble and finish genomes is a great advantage. However, the most common web tools, such as Galaxy [11], present several other functionalities beside of genome assembly, that can confuse the users and delay the finishing of genome assembly projects. Web tools with simple, objective and intuitive interface may help bioinformaticians not to worry about technical activities, as genome assembly, and focus in resolve more relevant questions.

In this context, we propose a web tool, called SIMBA (**SI**mple **M**anager for **B**acterial **A**ssemblies), which includes, through a friendly interface and easy installation, several scripts for analysis and data conversion, software for *de novo* assembly, construction of scaffolds and closing of gaps. SIMBA can be installed on a Linux server and accessed through a web browser over the Internet. The main contribution of SIMBA is an intuitive interface that guides the users to execute assembly process through simple and interactive pages.

## Methods

**Fig. 1:**
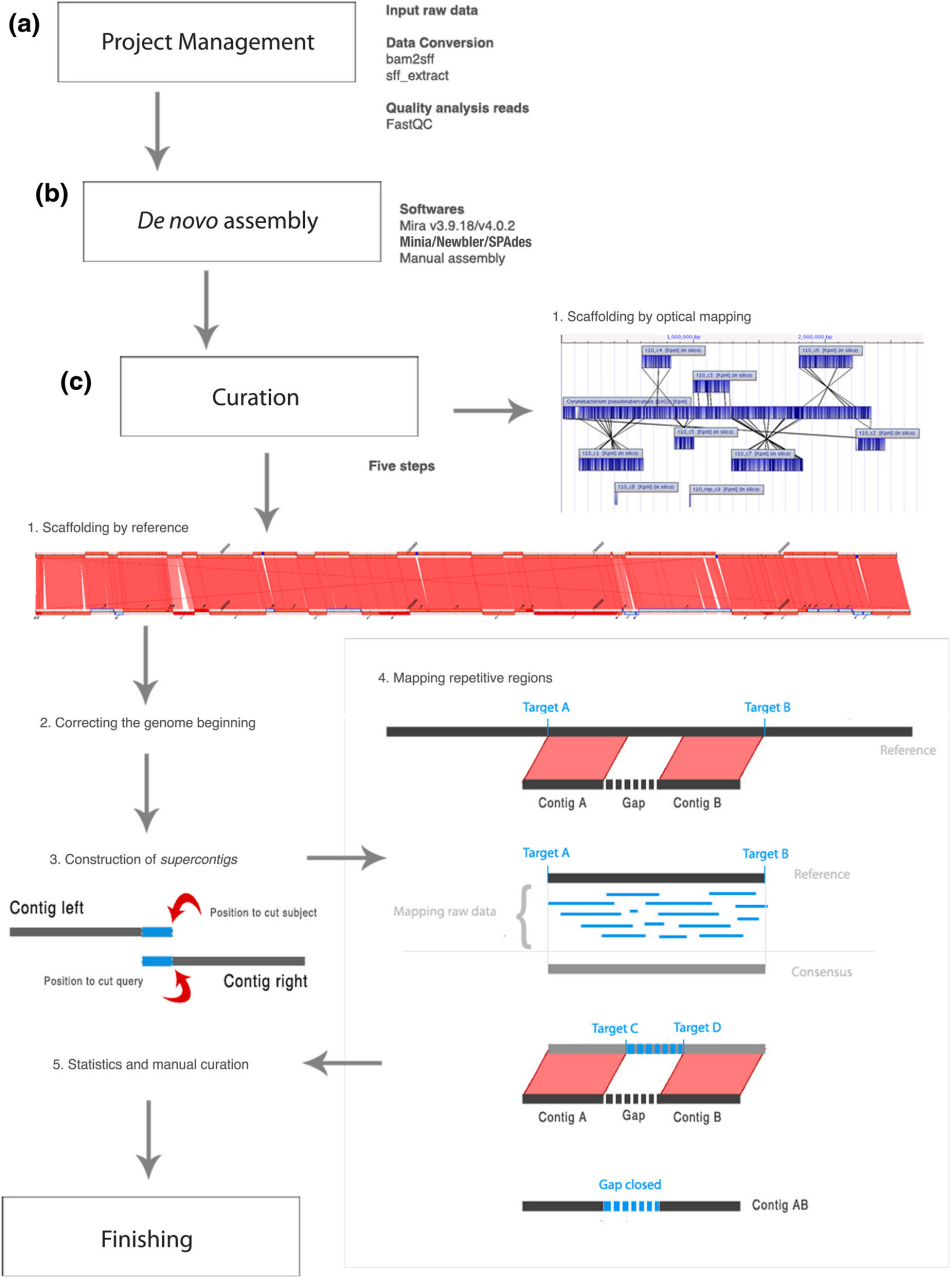
Workflow representing data fluxes in SIMBA’s process. **a** Projects management: creation and management of projects; and raw data conversion. **b** *De novo* assembly (Mira software – support to the assemblers Minia, Newbler and SPAdes). In this step the user can insert output files of *de novo* assembly yielded by external software. **c** Curation: This step has five subdivisions: 1) contigs orientation using CONTIGuator2 software by reference or by optical mapping report; 2) Start DNA correction based on reference genome; 3) merging neighbor contigs with overlap in flank regions using PHP parser and BLAST (blastn); 4) mapping 3,000 bp of flank regions of neighbor contigs (Contig A and Contig B) against reference genome; BLAST is used to determine the start and end position (targets A and B); the reference genome is trimmed at targets A and B positions; then the raw data is mapped using Mira4; the contigs A and B are mapped on consensus sequence obtained through raw data mapping; targets C and D are used to detect start and end position of specific GAP; the region is extracted, and the gap is closed. 5) Showing statistics about unknown nucleotides present in the genome and allow genome download for manual curation

SIMBA interface was developed using Laravel PHP framework (http://laravel.com), front-end framework Bootstrap (http://getbootstrap.com) and SQlite database (https://www.sqlite.org). The internal working process of SIMBA was divided into three modules: projects, assemblies and curation (Fig. 1).

### Projects

Projects module (Fig. 1a) shows a general vision of genome sequencing projects, in addition it allows data quality analysis and data format conversions. In this module, each genome assembly project is linked to a raw data file. As input files, SIMBA supports the extensions: BAM, SFF or FASTQ and XML. The user can convert the files through different customized programs and scripts, for example, extract a file in FASTQ and XML format from a file in SFF format. It is also possible to visualize read quality reports yielded by FastQC software (http://www.bioinformatics.babraham.ac.uk/projects/fastqc).

### Assemblies

SIMBA allows each project to make several attempts for *de novo* assembly in the Assemblies module (Fig. 1b). Therefore, SIMBA is integrated by default with assembly software Mira v.3.9.18 (Mira3) [12]. SIMBA also provides support to assemblers: Mira v.4.0.2 (Mira4), SPAdes [13], Newbler (http://www.454.com/products/analysis-software) and Minia [14]. Mira3 and Mira4 were configured with the parameters “job = genome,denovo,accurate parameters = −GE:not = 16 IONTOR_SETTINGS -AS:mrpc = 100”. Minia was configured with the parameters “k_mer = 31 and length genome = 2,500,000”. SPAdes was configured with the “--iontorrent” parameter. No parameter was applied to Newbler. The user can change the parameters. Considering the type of licensing of software, only the two Mira versions and SPAdes are enabled by default, requiring the user to register and download the other tools. The user also can import an assembly that was accomplished outside the application interface. SIMBA analyzes the results of the assemblies through scripts in Python, stores them in the SQLite database, and displays at the online interface. Moreover, the interface allows assembly quality analysis using QUAST software [15].

### Curation

The curation module (Fig. 1c), or assembly finishing module, was divided into five steps: (i) contigs scaffolding based on reference genome using CONTIGuator v2 software, or contigs orientation based on whole-genome mapping (WGM) report generated by the MapSolver software; (ii) setting start replication site of circular genome based on reference genome (for reference scaffolding); (iii) building of supercontigs by analyzing the ends of contigs’ neighbors through the Basic Local Alignment Search Tool (BLAST) [16]; (iv) mapping of raw data sequence in the reference genome to extract a consensus sequence as a strategy to solve repetitive regions, such as phages, plasmids, transposons and rRNA operons through MapRepeat software (for reference scaffolding); (v) displaying statistical reports about the presence or absence of nucleotides that were not determined.

Finally, the user can download the final genome file for manual curation with stand-alone tools, such as analysis of remaining gaps and evaluation of regions with sequencing errors to frameshifts correction. Soon after, the curated file with the final result can be imported into SIMBA and stored for future reference.

## Results and discussion

SIMBA presents several great functionalities, such as *de novo* assembly with different software, assembly quality evaluation, scaffolding by reference or by optical mapping, genome visualization, and gap closing through supercontigs construction. The user-friendly web interface allows that multiple users work in parallel. In the software compared, only Galaxy presented this advantage (Table 1). SIMBA is free and open source, and for simple genome assemblies presents as good functionalities as commercial software, such as CLC Genomics Workbench and Lasergene Suite (Table 1).

**Table 1:** Comparison among SIMBA and other software

	SIMBA	Galaxy	CLC	Lasergene
User-friendly interface	X	X	X	X
*De novo* assembly	X	X	X	X
*De novo* assembly with multiple algorithms	X	X		
Scaffolding by reference	X	X	X	X
Assembly quality evaluation	X			
Scaffolding by optical mapping	X			
Gap closing	X	X	X	X
Genome visualization	X			
Free and open source	X	X		
Web tool	X	X		
Multiple users in parallel	X	X		
Free sequence edition			X	X
Annotation support		X	X	X

After the development of the web tool, we used seven genomes to test the efficacy of SIMBA to manage bacterial assembly projects (Table 2). SIMBA was executed in an Apache web server (http://httpd.apache.org) installed in an operational system CentOS 6.4, 1 TB RAM, 30 TB hard disk and AMD processor with 64 cores. The genomes were obtained by NGS sequencing using Ion Torrent PGM 200 bp sequencing kit (400 bp kit for NCDO2118). For each project, BAM file with raw data was converted to SFF and extracted to FASTQ and XML format through the interface. The *de novo* assemblies were performed using Mira3, Mira4, Minia, Newbler and SPAdes. Some assemblies fail due to unknown reasons. The objective of this case study was not to compare assembly results, but certify the facility to perform assemblies with different software in SIMBA. The lowest number of contigs and the QUAST analysis were used to define which assembly would be used for the scaffolding step.

**Table 2:** Assemblies using SIMBA

Genome	NGS	Software	Contigs	Scaffolding method	Genome length (bp)	Total scaffolds	Reference
*Corynebacterium pseudotuberculosis* 258 (Cp258)	Ion PGM	Mira3	41	Optical mapping (enzyme *kpnI*)	2,369,817	15	Data not published.
		Mira4	56				
		Minia	675				
		Newbler	27				
		SPAdes	58				
*Corynebacterium pseudotuberculosis* 1002 (Cp1002)	Ion PGM	Mira3	9	Optical mapping (enzyme *kpnI*)	2,335,107	5	[18]
		Mira4	12				
		Minia	2,425				
		Newbler	10				
		SPAdes	15				
*Corynebacterium pseudotuberculosis* 12C (Cp12C)	Ion PGM	Mira3	25	Reference (*Corynebacterium pseudotuberculosis* PAT10)	2,337,451	9	[22]
		Mira4	62				
		Minia	-				
		Newbler	17				
		SPAdes	631				
*Corynebacterium pseudotuberculosis* VD57 (CpVD57)	Ion PGM	Mira3	11	Reference (*Corynebacterium pseudotuberculosis* 31)	2,337,177	6	[23]
		Mira4	15				
		Minia	1,146				
		Newbler	9				
		SPAdes	10				
*Corynebacterium ulcerans* FRC11 (FRC11)	Ion PGM	Mira3	-	Reference (*Corynebacterium ulcerans* 0102)	2,442,826	6	[24]
		Mira4	30				
		Minia	21^a^				
		Newbler	9				
		SPAdes	8				
*Corynebacterium ulcerans* 210932 (210932)	Ion PGM	Mira3	12	Reference (*Corynebacterium ulcerans* 0102)	2,484,335	2	[25]
		Mira4	-				
		Minia	343				
		Newbler	8				
		SPAdes	18				
*Lactococcus lactis subsp. lactis* NCDO 2118 (NCDO2118)	Ion PGM	Mira3	661	Reference (*Lactococcus lactis* KF147)	2,554,693	18	[26]
		Mira4	-				
		Minia	-				
		Newbler	43				
		SPAdes	-				

^a^Minia assembled only 8,097 bp of the genome

-The assembly fails for an unknown reason

The assembly completion was performed through two approaches: reference scaffolding (for strains Cp12C, CpVD57, FRC11, 210932 and NCDO2118) and optical mapping scaffolding (for strains Cp258 and Cp1002). For reference scaffolding, the templates were retrieved from on the NCBI database (https://www.ncbi.nlm.nih.gov/genome). For optical mapping scaffolding, it was necessary to construct restriction maps using Argus system (OpGen Inc, Gaithersburg, MD) Whole-genome mapping technique. This technique consists in (i) the extraction of chromosomal DNA of both strains; (ii) immobilization and in situ restriction digestion (it was used the enzyme *kpnI*); (iii) image capture and measurement of fragments; and (iv) map assembly and analysis using the MapSolver software [8]. The contigs order report was used in the step one of the curation module in SIMBA.

SIMBA produced a low number of scaffolds in all sequencing projects (Table 2). All the assemblies were finalized and the genomes were deposited as complete sequences, however manual curation using the software CLC Genomic Workbench 7 was necessary to close some gaps. SIMBA did not detect overlapping of contigs smaller than the word length of the blastn software or gaps without overlaps and information in the reference genome. CLC Genomic Workbench and other commercial software have functions to extend contigs and edit freely sequences, which allow the human intervention to close these types of gaps. However, this can imply in a higher number of mis-assemblies. SIMBA tries minimizing the number of mis-assemblies due to manual curation, for this reason SIMBA was not capable to close all gaps.

SIMBA provided an easy and fast method to help finishing the assemblies of Cp1002, Cp258, Cp12C, CpVD57, FRC11, 210932 and NCDO2118. SIMBA allowed other software analyzed the genomes, and also that the genomic data were stored when the assemblies were finished. Our study case shows evidences that SIMBA can be efficiently used for prokaryotic genome assemblies of Ion Torrent sequencing data. SIMBA can be used with other NGS data, such as data obtained by Illumina Sequencing Systems. SIMBA was designed and tested only to the Ion PGM sequencing technology, and there are no guarantees that SIMBA can manage Illumina data at this version with the same efficiency. SIMBA allows managing several projects in parallel and provides reports that help to analyze the assemblies.

**Fig. 2:**
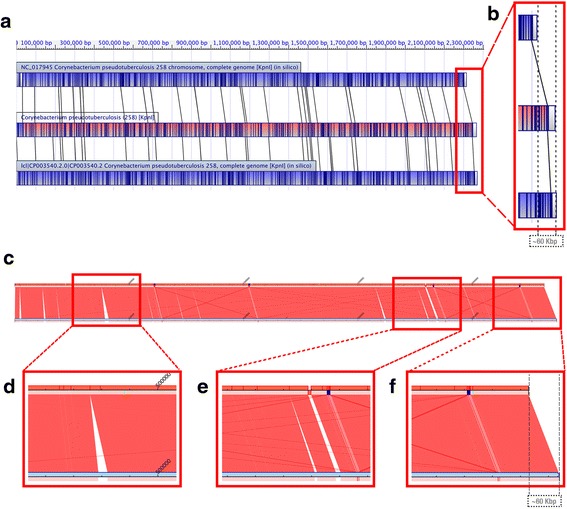
MapSolver and SIMBA visualizations of the comparison between the new and old assemblies of Cp258. **a** MapSolver alignment visualization among the whole-genome optical mapping (enzyme *kpnI*; the central barcode in the color red), the old assembly (NC_017945; the barcode above in the color blue), and the new assembly (performed by SIMBA; the barcode below also in the color blue). Dark blue lines in the barcodes represent restriction sites. Lines connecting barcodes represent similarity regions. **b** The old assembly (above) is ~60 Kbp smaller than the restriction map (center), that have a length near to the new assembly (below). **c** SIMBA visualization compares the Cp258 old assembly (horizontal line red above) with the new assembly (horizontal line light blue below). Red lines that connect the line above and the line below represent syntenic regions. The visualization shows: **d** regions undetected in the old assembly; **e** mis-assemblies in the old assembly; and (**f**) the length difference between the genomes. The visualization showed by SIMBA agrees with the MapSolver results. In addition, it gives more detailed information about the genome differences

The assemblies of Cp258 and Cp1002 showed differences with the original genomes. The new assembly of Cp258 (length: 2,369,817 bp) detected an increase of ~60 Kbp over the original genome (length: 2,314,404 bp; GenBank: NC_017945; [17]). The SIMBA’s genomic visualization confirmed and extended the MapSolver results, allowing a detailed comparison between the old assembly and the new assembly (Fig. 2). It also allowed that were detected insertion positions (Fig. 2d) and mis-assemblies (Fig. 2e). The use of WGM technique has allowed the discover of mis-assemblies in bacterial genomes, such as a large inversion in Cp1002 recently published [18]. WGM has been considered a strategy of high accuracy to finish assemblies [18–21]. However, MapSolver is not capable to generate scaffolds. SIMBA allows deal with contigs order reports of MapSolver, produces scaffolds based on the restriction positions, and thus, more accurate assemblies than based on reference genome.

## Conclusions

SIMBA is a simple and useful tool, which uses a friendly interface to abstract complex tasks of the bacterial genome assembly process. It can enable researchers with little or no experience in computer science to use it thereby reducing the execution time of tasks and the associated cost. SIMBA facilitates the data conversion process, sequencing and assemblies’ quality evaluation, running different assembly software, scaffolding of high accuracy with WGM, as well as comparison of multiple outcomes allowing the use of visual perception as a transforming element in assembly finishing processes. Another benefit of SIMBA lies in the centralization of fitting data in laboratories, allowing their managers to monitor numerous assembly projects simultaneously. Finally, SIMBA allows the construction of an institutional knowledge base for genome assembly and allows for collaborative access. We have as prospects for the future integration of annotation tools in the SIMBA interface.

## Availability and requirements

  • **Project name:** UFMG-SIMBA

• **Project home page:** http://ufmg-simba.sourceforge.net

• **Operating system(s):** Linux 64bit (Server), Platform independent (Client)

• **Programming languages:** PHP, Python

• **Other requirements:** NCBI-BLAST+, Biopython library, Apache Server

• **License:** GPL v3

• **Any restrictions to use by non-academics:** None

The documentation can be obtained in the project home page. We also make available a Linux virtual machine based on SIMBA installed (including demo genome assembly) at the same URL. The new assembly of Cp258 was deposited under GenBank assembly accession GCA_000263755.2.

## Abbreviations

Cp258: *Corynebacterium pseudotuberculosis* 258
Cp1002: *Corynebacterium pseudotuberculosis* 1002
Cp12C: *Corynebacterium pseudotuberculosis* 12C
CpVD57: *Corynebacterium pseudotuberculosis* VD57
FRC11: *Corynebacterium ulcerans* FRC11
210932: *Corynebacterium ulcerans* 210932
NCDO2118: *Lactococcus lactis subsp. lactis* NCDO 2118
WGM: Whole-genome mapping
